# Use of antipsychotics in Denmark 1997–2018: a nation-wide drug utilisation study with focus on off-label use and associated diagnoses

**DOI:** 10.1017/S2045796021000159

**Published:** 2021-04-06

**Authors:** M. Højlund, J. H. Andersen, K. Andersen, C. U. Correll, J. Hallas

**Affiliations:** 1 Department of Public Health, Clinical Pharmacology, Pharmacy, and Environmental Medicine, University of Southern Denmark, Odense, Denmark; 2 Mental Health Services Region of Southern Denmark, Department of Psychiatry Aabenraa, Aabenraa, Denmark; 3 Department of Clinical Research, Psychiatry, University of Southern Denmark, Odense, Denmark; 4 Mental Health Services Region of Southern Denmark, Department of Psychiatry Odense, Odense, Denmark; 5 Department of Psychiatry, The Zucker Hillside Hospital, Zucker School of Medicine at Hofstra/Northwell, New York, USA; 6 Department of Psychiatry and Molecular Medicine, Donald and Barbara Zucker School of Medicine at Hofstra/Northwell, Hempstead, NY, USA; 7 Department of Child and Adolescent Psychiatry, Charité Universitätsmedizin, Berlin, Germany

**Keywords:** Antipsychotics, off-label, pharmacoepidemiology, quetiapine

## Abstract

**Aims:**

Antipsychotics are primarily labelled for the treatment of severe mental illness and have documented clinical utility in certain neurological disorders or palliative care. However, off-label use of antipsychotics is common and increasing, and prior studies on antipsychotic utilisation have not specifically assessed users in neurology, palliative care or general practice. We aimed to explore diagnoses associated with antipsychotic use, treatment patterns and characteristics of users without diagnoses relevant to antipsychotic treatment.

**Methods:**

Population-based study identifiying all users of antipsychotics in Denmark (pop 5.7 mio.) 1997–2018 in the Danish National Prescription Register (DNPR). Possible indications for antipsychotic therapy were evaluated using in- and outpatient contacts from the DNPR. Users were divided hierarchically into six groups: severe mental disorders (schizophrenia, bipolar-spectrum disorders), chronic mental disorders (dementias, mental retardation, autism), other mental disorders (depression-spectrum, anxiety and personality disorders, etc.), selected neurological diseases, cancer and antipsychotic users without any of these diagnoses. This last group was characterised regarding demographics, antipsychotic use, health care utilisation and likely antipsychotic treatment initiator in 2018.

**Results:**

Altogether, 630 307 antipsychotic users were identified, of whom 127 649 had filled prescriptions during 2018. Users without diagnoses relevant to antipsychotic treatment comprised of the largest group (37%), followed by schizophrenia and bipolar-spectrum disorders (34%), other mental disorders (15%), dementia, autism and mental retardation (11%), cancer (2.2%) and neurological diagnoses (2.0%). Of 37 478 incident users in 2018, 39% had no diagnosis relevant to antipsychotic treatment, 7.9% had major depression, 7.7% neurotic/stress-related disorders and 7.5% dementia. Quetiapine was most commonly used, both overall (51%) and among users without diagnoses relevant to antipsychotic treatment (58%). Of 14 474 incident users in 2018 without diagnoses relevant to antipsychotic treatment, treatment was most likely initiated by a general practitioner (65%), with only 17% seeing a psychiatrist during the following year. As many as 18% of patients with adjustment disorders and 14% of those without relevant diagnoses for antipsychotic use, remained on antipsychotic treatment 5 years after their first prescription.

**Conclusions:**

Over one-third of antipsychotic users in Denmark did not have psychiatric, neurological or cancer diagnoses as possible indications for antipsychotic therapy. Many antipsychotics are initiated or prescribed in general practice, and a concerningly large subgroup without documented diagnoses relevant for antipsychotics continued to receive them. Rational prescribing, adequate side effect monitoring and further research into reasons for the observed antipsychotic use patterns and their risk–benefit ratio are needed.

## Introduction

Antipsychotics are generally labelled for treatment of severe mental disorders, such as schizophrenia, mania and bipolar depression. Other licensed indications can be insufficiently responding unipolar depression, autism and Tourette's syndrome. Furthermore, the use of antipsychotics can be clinically relevant in other psychiatric conditions that do not have a licensed indication, such as dementia, post-traumatic stress disorder or obsessive-compulsive disorder. Caution is warranted when using antipsychotics, as they are associated with a number of potentially serious adverse effects, including fatal arrhythmias, metabolic disturbances and extrapyramidal symptoms (Solmi *et al*., 2017; Stroup and Gray, 2018; Papola *et al*., 2019).

However, prior drug utilisation studies have found a considerable use of antipsychotics in other psychiatric conditions for which antipsychotics do not have an indication, including dementia, anxiety disorders and insomnia (Marston *et al*., 2014; Carton *et al*., 2015; Baandrup and Kruse, 2016). Furthermore, antipsychotics are also used in other medical specialties than psychiatry, e.g. for delirium (Marcantonio, 2017), for psychotic symptoms in epilepsy (Agrawal and Mula, 2019), treatment of headache disorders (Siow *et al*., 2005; Bendtsen *et al*., 2012), as antiemetics (Walsh *et al*., 2017) or in end-of-life care (Bush *et al*., 2017).

The dispensed quantity of antipsychotics has remained stable in Denmark over the past 10 years, while the prevalence of antipsychotic use has increased during the same period, indicating increasing low-dose use of antipsychotics (Danish Health Data Authority). The reasons for this increase are poorly understood. However, a pronounced decrease in the use of benzodiazepine analogues has been observed over the same period (Danish Health Data Authority), which might have been replaced, at least partly, by low-dose use of antipsychotics acting as anxiolytics or hypnotics. The quite low average quantities dispensed to each user lends some support to this hypothesis (Højlund *et al*., 2019).

Studies addressing the underlying drivers of antipsychotic utilisation are scarce (Olfson *et al*., 2012; Baandrup and Kruse, 2016), and prior studies on overall antipsychotic utilisation commonly lack information on associated diagnoses (Hálfdánarson *et al*., 2017; Højlund *et al*., 2019), or were confined to patients with psychiatric diagnoses or contacts (Olfson *et al*., 2012; Baandrup and Kruse, 2016). Thus, these studies did not assess the entire population of users treated in general practice, private psychiatric practice or other medical specialties than psychiatry.

The aim of this study was to analyse current patterns and long-term trends in antipsychotic utilisation, including associated diagnoses, treatment persistence and characteristics of users without diagnoses relevant to antipsychotic treatment.

## Method

### Study design and data sources

We conducted a nation-wide drug utilisation study to explore current patterns and long-term trends in antipsychotic use by identifying all Danish residents who filled a prescription for an antipsychotic between 1 January 1997 and 31 December 2018 in the Danish Register of Medicinal Product Statistics (DRMPS) (Pottegård *et al*., 2017). Antipsychotics were defined as all medications within the World Health Organization (WHO) Anatomical Therapeutic Chemical Classification (ATC) group N05A (WHOCC-ATC/DDD Index), excluding lithium (ATC N05AN01). Preparations within ATC-group N05A are only available on prescription, and all dispensing at community pharmacies is recorded in the DRMPS.

Prescription data were then linked, using civil registration numbers, to information on psychiatric diagnoses from the Danish National Patient Register (DNPR) (Lynge *et al*., 2011) and the Danish Psychiatric Central Research Register (DPCRR) (Mors *et al*., 2011), and to information on health care utilisation from the National Health Insurance Services Register (NHISR) (Andersen *et al*., 2011). DRMPS contains information on all prescriptions dispensed at Danish community pharmacies from 1995 onwards. DNPR contains information on hospital contacts and diagnoses from all admissions or outpatient contacts to Danish hospitals since 1977 and 1995 respectively. DPCRR contains information on admissions to psychiatric hospitals from 1970 and outpatient contacts to psychiatric facilities from 1995. NHISR contains information on all contacts to general practitioners and practicing specialists from 1990 onwards and is based on invoices to the region health administrations. Virtually, all health care in Denmark is publicly funded, and thus captured in these registers. An overview of the underlying data sources is provided in online Supplementary Appendix 1.

### Outcome measures and statistical analysis

We analysed the data in four dimensions: (1) overall drug use statistics, (2) diagnoses associated with antipsychotic use, (3) characterisation of users without diagnoses relevant to antipsychotic treatment and (4) treatment persistence for selected subgroups.

#### Overall drug use statistics

We calculated 1-year prevalence as the total number of users divided by the population base, and incidence as the number of new antipsychotic users (i.e. users without antipsychotic prescriptions in the preceding year) divided by the population base. Mean dose was calculated as the total amount of antipsychotic sold divided by the number of users for that antipsychotic divided by 365 days per year, resulting in the average daily dose for all users of that antipsychotic (unit: DDD/user/day). To assess overall differences in treatment duration, we calculated a duration index as *P*/(1 − *P*) × *I* (where *P* is the prevalence and *I* is the incidence for the specific antipsychotic) (Hallas and Støvring, 2006). Prevalent users were defined as users with antipsychotic prescriptions in the preceding year and incident users as users without prescriptions in the preceding year. High duration indices above 1 indicate a retention of users (i.e. continuous or recurrent treatment). To assess skewness in antipsychotic consumption, we calculated 1st and 50th percentiles as the proportion of antipsychotic sales accounted for by the 1 and 50% most intensive users (Hallas and Støvring, 2006).

#### Diagnoses associated with antipsychotic use

Antipsychotic users were divided into six groups based on occurrence of in- or outpatient diagnoses in the DNPR/DPCRR. We used an appropriateness hierarchy based on main indications for antipsychotic therapy, followed by other relevant chapters of the WHO International Statistical Classification of Diseases and Related Health Problems, 10th revision (ICD-10) (see online Supplementary Appendix 2 for specific codes):

- Group 1 ‘Severe mental disorders’: users diagnosed with schizophrenia, schizoaffective disorder, other delusional disorders, mania or bipolar affective disorder.
- Group 2 ‘Chronic mental disorders’: users diagnosed with dementias, mental retardation, autism and no record of diagnoses in group 1.
- Group 3 ‘Other mental disorders’: users with other psychiatric diagnoses (e.g. major depression, anxiety disorders or personality disorders) and no record of diagnoses in groups 1 and 2.
- Group 4 ‘Neurological diagnoses only’: users with selected neurological diagnoses where antipsychotic treatment might be relevant (e.g. Parkinson's disease), and no record of diagnoses in groups 1–3.
- Group 5 ‘Cancer diagnoses only’: users with diagnosis of a malignant neoplasm and no record of diagnoses in groups 1–4 suggesting use in palliative care.
- Group 6 ‘No relevant diagnosis’: users with no record of diagnoses in groups 1–5.

Users were assigned to group 1 or 2 if they had any occurrence of these diagnoses in registers between register inception (1997 for inpatient diagnoses and 1995 for outpatient diagnoses, see online Supplementary Appendix 1) and their first antipsychotic prescription that year. All other users were assigned to a group based on occurrence of diagnoses within 6 months before or after their first antipsychotic prescription that year. We used a 6-month window to allow subsequent diagnoses to be associated with the current prescription in incident users, and to capture outpatient visits that were separated in time from prescription redemptions. The groups (and subgroups) were hierarchical, such that an individual would be assigned as belonging to the lowest possible group (or subgroup) number. For all years in the study period, we defined prevalent users as users with antipsychotic prescriptions in the preceding calendar year and incident users as users without prescriptions in the preceding year. Additionally, we conducted a sensitivity analysis extending the assessment period from 1 to 2 and 5 years, respectively, before 2018 to explore the proportion of ‘intermittent users’.

#### Characterisation of antipsychotic uses without diagnoses relevant to antipsychotic treatment

Users in group 6 were characterised in terms of demographics, antipsychotics used, number of prescriptions, number of antipsychotics used, total amount redeemed, concurrent use of psychotropic medications, somatic comorbidity, first prescriber (incident users only) and health care utilisation (incident users only). The use of other psychotropic medications was assessed as prescriptions of drugs listed in online Supplementary Appendix 3 within 3 months before or after the first antipsychotic prescription in 2018. Somatic co-morbidities were assessed as any occurrence of the diagnoses or prescriptions listed in online Supplementary Appendix 3 before the first antipsychotic prescription in 2018. Incident users without hospital contacts were linked with NHISR to assess health care use outside the hospital system. To assess the likely first prescriber, we evaluated health care contacts in NHISR 14 days prior to the first antipsychotic prescription as most patients will fill prescriptions within few days after the prescription was issued (Pottegård *et al*., 2014). We categorised health care contacts as ‘general practitioner’, ‘psychiatrist’ and ‘neurologist’. If the user had been in contact with both a general practitioner and a specialist within this 14-day period, conservatively, the latter was assigned as the likely first prescriber. Health care utilisation in general was assessed as any contact in NHISR during 2018 with a psychiatrist, neurologist or a general practitioner only.

#### Treatment persistence for selected subgroups

We estimated persistence of antipsychotic use for individuals with schizophrenia/schizoaffective disorder, dementias, adjustment disorders and no relevant diagnoses using ‘proportion of patients covered’ (PPC) as described by Rasmussen *et al*. (2018). These groups were chosen, as they are expected to represent different treatment patterns, e.g. long-term treatment in schizophrenia, episodic treatment in dementia and short-term treatment in adjustment disorders. Treatment persistence was calculated as the proportion of new users within subgroup who were covered by their latest prescription, conservatively assuming the use of one tablet per day. In contrast to traditional drug survival analyses the PPC-approach allows patients to re-enter in analyses as treated when they redeem new prescriptions. Thereby, PPC is less sensitive to assumptions about the treatment period that should be assigned to a single prescription (Rasmussen *et al*., 2018).

### Other

Data management and analyses were conducted with STATA MP release 15.1 (StataCorp, College Station, TX, USA). Approval for data access was obtained from the Danish Health Data Authority. According to Danish law, no ethical approval or informed consent is needed for purely register-based studies.

## Results

We identified a total of 19 092 613 antipsychotic prescriptions in the DRMPS from 1997 to 2018, filled by 630 307 individuals. The median number of prescriptions per individual was 4 (total range: 1–2465, interquartile range: 1–24), and the proportion of individuals with >1 antipsychotic prescription was 71%. The prevalence of antipsychotic use increased by 5.3% from 20.9 users/1000 inhabitants in 1997 to 22.1 users/1000 inhabitants in 2018.

### Overall antipsychotic use statistics

In 2018, the ten most prescribed antipsychotics (in terms of users) accounted for 91% of the total volume sold: quetiapine (51% of all users), olanzapine (14%), risperidone (13%), chlorprothixene (11%), aripiprazole (9.7%), haloperidol (6.2%), zuclopenthixol (3.3%), levomepromazine (3.1%), flupentixol (3.0%) and clozapine (2.7%) (Table 1). The highest rates of new users were observed for quetiapine and haloperidol with 3.92 and 1.22 new users per 1000 inhabitants, respectively. The highest 50th percentiles were observed for haloperidol, quetiapine, levomepromazine, risperidone and chlorprothixene, whereas the highest duration indices were observed for sulpiride, clozapine, perphenazine, sertindole and zuclopenthixol (Table 1).

**Table 1.** Drug statistics for all marketed antipsychotic drugs in Denmark in 2018 (population base: 5 781 190 inhabitants)

Antipsychotic drug (ATC)	DDD (oral use, mg)	Total number of users	One-year prevalence (users/1000 inhabitants)	Incidence rate (users/1000 inhabitants/year)	Volume sold (1000 DDD)	Mean dose (DDD/user/day)	Duration index^a^	1st percentile^b^ (% of total volume)	50th percentile^b^ (% of total volume)
All antipsychotics	–	127 649	22.08	6.48	21 286	0.46	3.5	10.0	94.5
Quetiapine (N05AH04)	400	64 946	11.23	3.92	6033	0.25	2.9	11.1	93.3
Olanzapine (N05AH03)	10	17 554	3.04	0.94	5293	0.83	3.3	5.5	88.8
Risperidone (N05AX08)	5	16 056	2.78	0.95	1906	0.33	2.9	7.1	90.7
Chlorprothixene (N05AF03)	300	14 028	2.43	0.85	613	0.12	2.8	10.3	89.7
Aripiprazole (N05AX12)	15	12 357	2.14	0.66	2808	0.62	3.3	4.3	85.0
Haloperidol (N05AD01)	8	7963	1.38	1.22	505	0.17	1.1	20.0	96.6
Zuclopenthixol (N05AF05)	30	4224	0.73	0.10	767	0.50	7.5	6.4	87.8
Levomepromazine (N05AA02)	300	4017	0.69	0.19	118	0.08	3.7	15.2	92.8
Flupentixol (N05AF01)	6	3809	0.66	0.15	282	0.20	4.3	18.8	87.9
Clozapine (N05AH02)	300	3403	0.59	0.06	1113	0.90	9.2	3.3	76.9
Paliperidone (N05AX13)	6	1523	0.26	0.07	587	1.06	4.0	3.4	78.2
Ziprasidone (N05AE04)	80	1066	0.18	0.03	441	1.13	7.3	4.0	81.6
Amisulpride (N05AL05)	400	720	0.13	0.03	176	0.67	3.6	4.8	86.4
Pimozide (N05AG02)	4	558	0.10	0.01	103	0.51	7.0	7.6	83.3
Perphenazine (N05AB03)	30	537	0.09	0.01	253	1.29	9.1	5.2	73.2
Lurasidone (N05AE05)	60	371	0.06	0.05	65	0.48	1.4	6.1	89.3
Prochlorperazine (N05AB04)	100	313	0.05	0.02	7	0.06	3.0	8.2	85.2
Sulpiride (N05AL01)	800	311	0.05	<0.01	75	0.67	17.4	5.0	77.8
Pipamperone (N05AD05)	200	222	0.04	0.01	26	0.32	4.7	7.8	88.4
Sertindole (N05AE03)	16	247	0.04	0.01	72	0.80	8.3	3.1	73.9
Melperone (N05AD03)	300	198	0.03	0.01	9	0.13	2.8	8.0	85.5
Periciazine (N05AC01)	50	170	0.03	<0.01	21	0.34	6.2	7.2	87.8
Asenapine (N05AH05)	20	25	<0.01	<0.01	4	0.47	5.7	–	–
Cariprazine (N05AX15)	3	(<5)	<0.01	<0.01	<1	0.04	–	–	–

ATC, Anatomic Therapeutic Chemical Classification System (who.int/classifications/atcddd/en); DDD, World Health Organization defined daily dose; ICD-10, World Health Organization International Statistical Classification of Diseases and Related Health Problems 10th revision (icd.who.int/browse10/2019/en).

a Duration index represents ratio between numbers of prevalent users and incident users.

b 1st and 50th percentiles describe the total amount (in percent) consumed by the 1 and 50% of users with the highest annual consumption, e.g. 50% of quetiapine users consume 93% of the dispensed amount of quetiapine, and the remaining 50% of users consumes 7%.

### Diagnoses associated with antipsychotic use

**Fig. 1.**
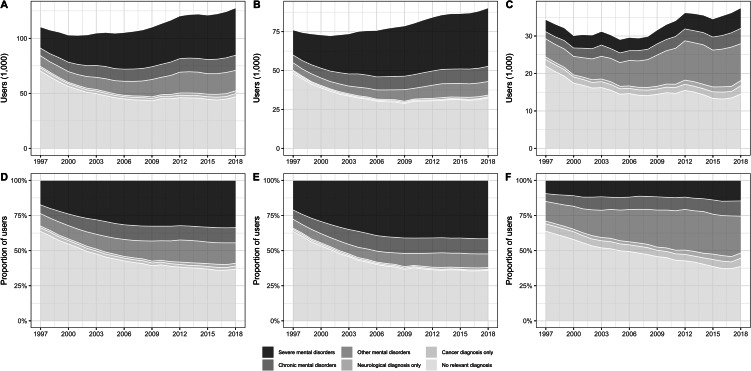
Development in total number of users by diagnostic groups and the proportion of users by diagnostic groups, 1997–2018 for all (A + D), prevalent (B + E) and incident users (C + F).

Since 1997, the proportion of users with severe mental disorders increased and the proportion of users without relevant diagnoses decreased (Fig. 1). The proportion of users in 2018 without severe mental disorders was 66% (84 716, Table 2).

**Table 2.** Diagnoses associated with antipsychotic use in Denmark, 2018

	All users			Prevalent users			Incident users			
ICD-10 diagnosis	*N*	% (all users)	% (sub-group)	*N*	% (all users)	% (sub-group)	*N*	% (all users)	% (sub-group)	Duration index^a^
All users	127 649	100	.	90 171	100	.	37 478	100	.	3.5
Severe mental disorders	42 933	34	100	37 464	42	100	5469	15	100	7.9
Schizophrenia and schizoaffective disorder (F20 + 25)	22 931	18	53	20 789	23	55	2142	5.7	39	10.8
Other delusional disorders (F22–24, 26–29)	10 859	8.5	25	8883	9.9	24	1976	5.3	36	5.5
Mania and bipolar affective disorder (F30–31)	9143	7.2	21	7792	8.6	21	1351	3.6	25	6.8
Chronic mental disorders	13 836	11	100	9754	11	100	4082	11	100	3.4
Dementia (F00–03, G30–31)	7292	5.7	53	4483	5	46	2809	7.5	69	2.6
Mental retardation and autism (F70–79 + 84)	6544	5.1	47	5271	5.8	54	1273	3.4	31	5.2
Other mental disorders^b^	18 594	15	100	8744	9.7	100	9850	26	100	1.9
Organic mental orders (excl. dementia) (F04–09)	1638	1.3	8.8	480	0.53	5.5	1158	3.1	12	1.4
Psychoactive substance use (F10–19)	4014	3.1	22	2243	2.5	26	1771	4.7	18	2.3
Schizotypal disorder (F21)	536	0.42	2.9	306	0.34	3.5	230	0.61	2.3	2.3
Psychotic depression (F32.3, 33.3)	459	<1	2.5	202	<1	2.3	257	<1	2.6	1.8
Depressive episode (F32.0-0.2)	2172	1.7	12	701	<1	8	1471	3.9	15	1.5
Recurrent depressive episode (F33.0-0.2)	2714	2.1	15	1535	1.7	18	1179	3.1	12	2.3
Phobias and other anxiety disorders (F40–41)	1792	1.4	9.6	772	0.86	8.8	1020	2.7	10	1.8
Obsessive-compulsive disorder (F42)	250	0.2	1.3	126	0.14	1.4	124	0.33	1.3	2.0
Reaction to severe stress (F43)	2927	2.3	16	1238	1.4	14	1689	4.5	17	1.7
Acute stress reaction (F43.0)	302	0.24	1.6	94	0.1	1.1	208	0.55	2.1	1.5
Post-traumatic stress disorder (F43.1)	987	0.77	5.3	531	0.59	6.1	456	1.2	4.6	2.2
Adjustment disorders (F43.2)	1048	0.82	5.6	398	0.44	4.6	650	1.7	6.6	1.6
Dissociative disorders (F44)	21	<0.1	0.11	16	<0.1	0.18	5	<0.1	<0.1	4.2
Somatoform disorders (F45)	60	<0.1	0.32	32	<0.1	0.37	28	<0.1	0.28	2.1
Eating disorders (F50)	158	0.12	0.85	84	<0.1	0.96	74	0.2	0.75	2.1
Nonorganic sleep disorders (F51)	52	<0.1	0.28	19	<0.1	0.22	33	<0.1	0.34	1.6
Specific and mixed personality disorders (F60–61)	960	0.75	5.2	563	0.62	6.4	397	1.1	4	2.4
Hyperkinetic disorder (F90)	377	0.3	2	191	0.21	2.2	186	0.5	1.9	2.0
Selected neurological diagnoses only^b^	2494	2	100	1108	1.2	100	1386	3.7	100	1.8
Encephalitis (G04–05)	9	<0.1	0.36	0	–	–	9	<0.1	0.65	.
Huntington's disease (G10)	11	<0.1	0.44	11	<0.1	0.99	0	–	–	.
Parkinson's disease (G20)	370	0.29	15	211	0.23	19	159	0.42	11	2.3
Multiple sclerosis (G35)	95	<0.1	3.8	59	<0.1	5.3	36	<0.1	2.6	2.6
Epilepsy (G40–41)	288	0.23	12	143	0.16	13	145	0.39	10	2.0
Migraine and other headache syndromes (G43–44)	311	0.24	12	136	0.15	12	175	0.47	13	1.8
Cerebrovascular disease (G45–46)	128	0.1	5.1	68	<0.1	6.1	60	0.16	4.3	2.1
Sleep disorders (G47)	1015	0.8	41	385	0.43	35	630	1.7	45	1.6
Hydrocephalus (G91)	47	<0.1	1.9	17	<0.1	1.5	30	<0.1	2.2	1.6
Cancer diagnosis only	2869	2.2	.	652	0.72	.	2217	5.9	.	1.3
No relevant diagnosis	46 923	37	.	32 449	36	.	14 474	39	.	3.3

ICD-10, World Health Organization International Statistical Classification of Diseases and Related Health Problems 10th revision (icd.who.int/browse10/2019/en).

a Duration index represents ratio between prevalent users and incident users.

b Categories with few users have been left out due to confidentiality issues. Therefore, the displayed categories or subcategories does not necessarily add up to totals.

Antipsychotic use in chronic mental disorders accounted for 11% (13 836) of all users in 2018, with 69% (2809) of incident users in this group being individuals with dementia (Table 2). Antipsychotic use in other mental disorders was the third largest group among all users with 15% (18 594), and the second largest group among incident users in 2018 with 26% (9850, Table 2). Especially, the number of incident antipsychotic users belonging to other mental disorders increased considerably from 1997 onwards. Increasing antipsychotic use in affective disorders (excluding bipolar disorder) and neurotic or stress-related disorders was the underlying driver for this increase among both incident and prevalent users (online Supplementary Figs 1–3).

Overall, antipsychotic use in individuals with neurological or cancer diagnoses accounted only for a minor proportion of all users (2.0 and 2.2%, respectively, corresponding to 2494 and 2869 individuals). However, the proportion of incident users was considerably higher with 3.7 and 5.9% of all incident users, respectively (1386 and 2217 individuals, Table 2). In 2018, antipsychotic use in sleep disorders was the largest subgroup among neurological disorders, followed by use in Parkinson's disease, epilepsy and headache disorders. The antipsychotic use in sleep disorders increased from 28 individuals in 1997 to 1015 in 2018, whereas the number of antipsychotic users in other neurological disorders remained relatively stable (online Supplementary Figs 1–3).

Extending the assessment period for ‘incident use’ to 2 and 5 years prior to 2018, reduced the number of incident users in all categories, suggesting a subgroup of intermittent users in every category (online Supplementary Table 1). Overall, 21% of the ‘incident users’ in 2018 have had prescriptions of antipsychotics within the preceding 5 years. Individuals with severe mental disorders had the largest proportion of users with prior prescriptions of antipsychotics within 5 years (47%), whereas this proportion was 3–21% for the remaining groups (online Supplementary Table 1). Importantly, the proportion of users without diagnoses relevant to antipsychotic treatment remained the same (79%), but the absolute number was lower when extending the assessment period to 5 years (11 482 *v*. 14 474 users without prior antipsychotic prescriptions within 5 years and 1 year, respectively).

### Diagnoses associated with use of specific antipsychotics

**Fig. 2.**
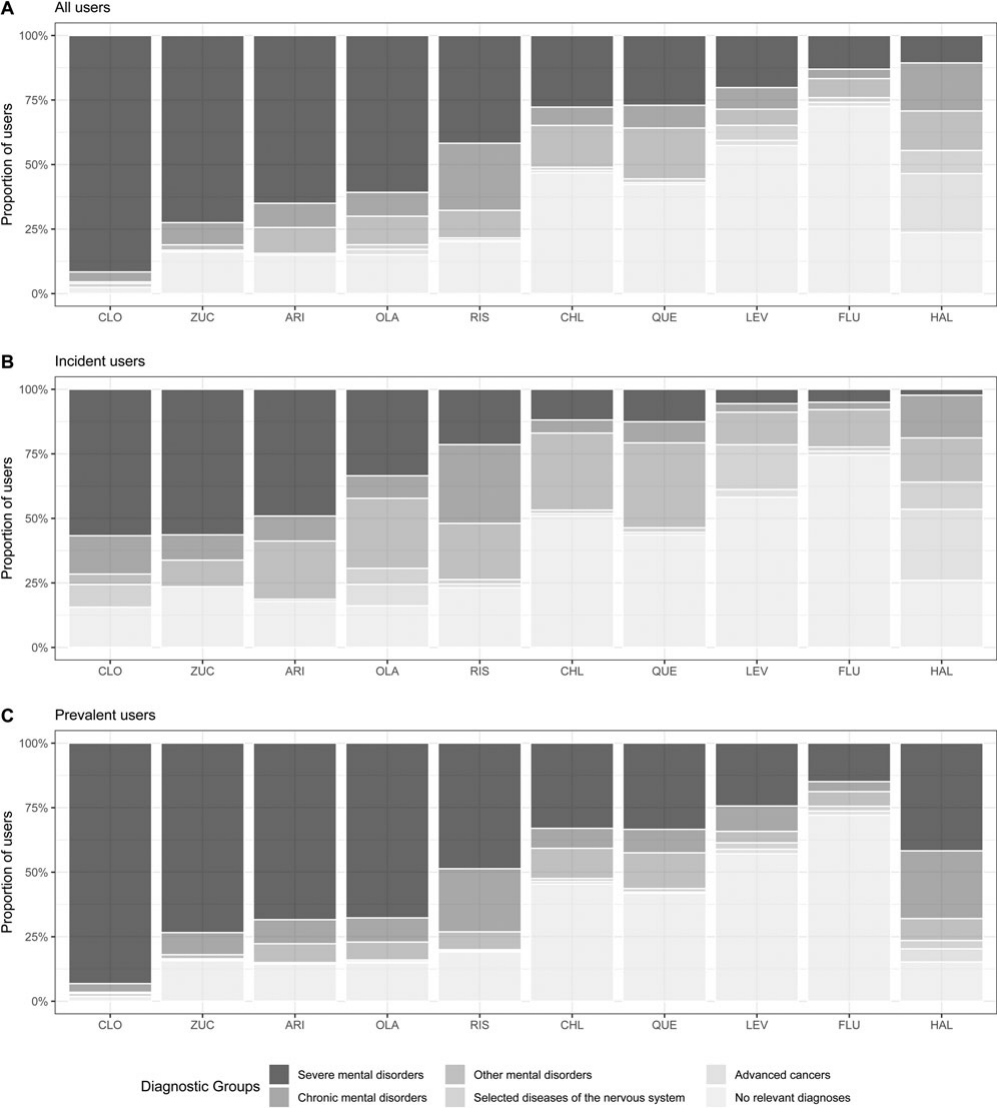
Proportion of users by diagnostic subgroups for commonly used antipsychotics, Denmark 2018.

In 2018, antipsychotics, such as clozapine, zuclopenthixol, aripiprazole and olanzapine, were predominantly used by individuals with severe mental illness (61–91% of users), while flupentixol, levomepromazine, chlorprothixene and quetiapine had high proportions of antipsychotic users without relevant diagnoses (47–72% of users). The proportion of users in each diagnostic group by commonly used antipsychotics can be seen in Fig. 2, and the total number of users is displayed in online Supplementary Table 2.

### Characteristics of users without diagnoses relevant to antipsychotic use

In this group, quetiapine was the most commonly used antipsychotic (58% of users) followed by chlorprothixene (14%). Most users in this group would use only one antipsychotic (93%), fill three or more prescriptions (60%) and use ⩽90 DDD (80%) (online Supplementary Table 3).

Of the 14 474 incident antipsychotic users in this group during 2018, only 12% had seen a practicing psychiatrist in the 14 days preceding their use of an antipsychotic. Furthermore, only 17% had seen a practicing psychiatrist at any time during 2018, and most antipsychotic users in this group (80%) had only been in contact with a general practitioner and had no relevant diagnosis in hospital registers within 6 months before or after their first antipsychotic prescription (online Supplementary Table 3).

A general practitioner was the initial prescriber in 65% of incident antipsychotic users in this group without diagnoses relevant to antipsychotic use (online Supplementary Table 3). For quetiapine users, this proportion was 68% and for chlorprothixene users it was 72%, whereas the numbers were considerably lower for users of other antipsychotics (online Supplementary Table 4). Haloperidol use in this group was predominantly by those aged 80 or more (72%), and for short-term use (70% with only one prescription) (online Supplementary Table 4). Median starting years early in the study period, indicating long-term use, was seen for flupentixol, levomepromazine and zuclopenthixol (2003, 2003 and 1995 respectively) (online Supplementary Table 4).

### Treatment persistence

**Fig 3.**
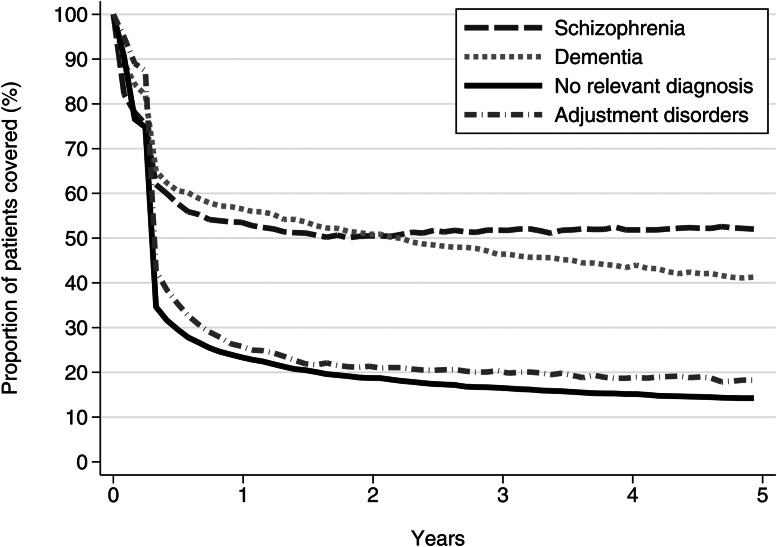
Duration of antipsychotic treatment measured by ‘PPC’ for selected subgroups.

Most antipsychotic users stopped their treatment within 6 months of first prescription. However, 57% of patients with dementia were still in treatment after 1 year, and 41% were still in treatment after 5 years. Among patients with adjustment disorders and those without relevant diagnoses, 18 and 14%, respectively, remained on antipsychotic treatment 5 years after their first prescription (Fig. 3).

## Discussion

The main findings of this nation-wide, 22-year antipsychotic utilisation study in 630 307 individuals filling 19 092 613 antipsychotic prescriptions are: (1) off-label antipsychotic use was highly prevalent; (2) most incident users were either diagnosed with non-severe mental illness or had no record of diagnoses relevant to antipsychotic treatment; (3) both overall and among patients without relevant diagnoses for antipsychotic use, quetiapine, used at low doses, was most frequently prescribed; (4) general practitioners most likely initiated antipsychotic treatment in users without relevant diagnoses for antipsychotic use and (5) long-term antipsychotic treatment was common in individuals with dementia, adjustment disorders and those without relevant diagnoses for antipsychotic use.

The increasing prevalence of antipsychotic use was driven by an increasing number of users in most diagnostic groups, although the number of users without relevant diagnoses for antipsychotic use decreased from 1997 to 2018. The finding of a considerable use of antipsychotics outside severe mental disorders is in line with prior drug utilisation studies from Denmark, France, the United Kingdom and the United States (Olfson *et al*., 2012; Marston *et al*., 2014; Baandrup and Kruse, 2016; Montastruc *et al*., 2018). The main addition of this study is the comprehensive evaluation of diagnoses associated with the use of antipsychotics in psychiatry as well as other medical specialties, including general practice.

One notable finding is that the number of incident users with non-severe mental disorders increased considerably over the study period. Some of these individuals might have diagnoses that, at some point, may benefit from off-label use of antipsychotics (e.g. anxiety disorders, borderline personality disorder, obsessive compulsive disorder and post-traumatic stress disorder) (Ingenhoven and Duivenvoorden, 2011; Liu *et al*., 2014; Slee *et al*., 2019; Zhou *et al*., 2019), or be in the process of psychiatric evaluation and eventually are diagnosed with severe mental illness. However, the substantial number of individuals and the variety of associated psychiatric diagnoses could suggest that the threshold for prescribing antipsychotics has decreased during the study period. A related finding is the large proportion of users without any record of psychiatric, neurological or cancer diagnoses in the registers. Here, evaluation of health care contacts found that most new users had not been evaluated by a psychiatrist or been in contact with a psychiatric emergency room. This finding suggests that the antipsychotic treatment was most likely initiated by a general practitioner for a condition that did not require specialised psychiatric evaluation or treatment.

A considerable proportion of users with dementia diagnoses continued long-term antipsychotic treatment, although this practice is not recommended due to e.g. increased risk of stroke and death (Douglas and Smeeth, 2008; Kales *et al*., 2012). The same pattern, although for a smaller proportion of patients, was observed for users with adjustment disorders or no diagnosis relevant for antipsychotic use in the registers. This pattern might reflect the use of antipsychotics as anxiolytics or hypnotics instead of benzodiazepines or benzodiazepine-related medications (Anderson and Vande Griend, 2014; Gjerden *et al*., 2017). However, the reasons for such continuous use and the relevance of deprescribing efforts should be investigated further.

The predominant use of quetiapine is important for several reasons: it is by far the most commonly prescribed antipsychotic in Denmark in 2018 filled by 51% of all users. In 2018, 42% of all quetiapine users had no record of diagnoses relevant to antipsychotic treatment, which were 27 447 individuals in total. Of these, 82% would redeem small quantities of quetiapine (<90 DDD/year) indicating low-dose/off-label use. This wide-spread use of quetiapine might be problematic as safety is not thoroughly evaluated with the use of quetiapine in low dose. However, prior observational studies have indicated increased risk of metabolic disturbances (Carr *et al*., 2016), fall-related injuries (Tapiainen *et al*., 2020), stroke (Correll *et al*., 2015) and all-cause mortality (Gerhard *et al*., 2020; Reutfors *et al*., 2020) with the use of quetiapine in individuals without severe mental disorders.

A major strength of the current study is its data sources, which are nation-wide and allow long-term follow-up. In Denmark, virtually all health care is publicly funded, especially for the investigated specialties (psychiatry, neurology, oncology and general practice), and thus captured in the DNPR or NHISR. Furthermore, all prescriptions filled at community pharmacies are recorded in DRMPS and use at long-term care facilities (e.g. nursing homes) are also included and individually referable.

Limitations of the current analyses must be acknowledged: first, the specific indication for antipsychotic therapy is not recorded in registers. This point is especially relevant for users treated in general practice or by specialists who do not report diagnostic information in the reimbursement process. Regarding the latter group, there are about 150–200 office-based psychiatrists (including child and adolescent psychiatrists) in Denmark who treat approximately 20% of the patients with psychiatric disorders (Mors *et al*., 2011). However, psychiatric disorders that require antipsychotic treatment are generally not treated solely by such practitioners and will likely generate in- or outpatient diagnoses in hospital registers. An exception from this rule could be individuals with bipolar affective disorder, depression or obsessive-compulsive disorder. To strengthen the appropriateness evaluation, we extended the evaluation of diagnoses in groups 1 and 2 to any occurrence between register inception and 6 months after the first prescription of an antipsychotic. Otherwise, individuals with e.g. schizophrenia, bipolar affective disorder, dementia or intellectual disabilities and no recent hospital contacts would lead to overestimation of other groups. Still, the possibility remains that some individuals would only have records of diagnoses in group 1 or 2 before DNPR inception in 1977/1995, that we were thus not able to evaluate (e.g. individuals with bipolar affective disorder on maintenance treatment with antipsychotics treated in office-based psychiatry). Second, the use of a 6-month window in the classification process is somewhat arbitrary. A wider window could direct attention away from the disorder associated with the relevant antipsychotic prescription (especially, for incident users), whereas a narrower window could ignore relevant information and result in overestimation of group 6. Third, we have to acknowledge that all diagnostic codes have imperfect sensitivity, i.e. we may have overlooked some conditions that would justify the use of antipsychotics e.g. that antipsychotic treatment was initiated in office-based psychiatry where diagnostic information is not accessible, or on the basis of advice from a specialist to e.g. a general practitioner. However, diagnoses of severe mental disorders as schizophrenia will most likely lead to hospital contacts at some point and high validity have been demonstrated for the schizophrenia diagnosis in Danish registers (Uggerby *et al*., 2013). Fourth, we had no data on which other non-pharmacologic or pharmacologic treatments were tried first and which may have failed. Fifth, prescribed daily doses are not available in the registers. Sixth, exact prescribed daily doses are not available in the registers and the utilised DDD method does not ensure fully equivalent dose levels for each individual antipsychotic. Finally, results are limited to Denmark, and may not generalise to other countries and health care settings.

Despite these limitations, results from this relatively large descriptive study indicate that a considerable number of users have no clear indication for antipsychotic therapy. Although off-label use might be warranted in some cases, attention should be given to enhance the rational use of antipsychotics that can have considerable adverse effects (Solmi *et al*., 2017; Papola *et al*., 2019). Initiatives which focus on rational prescribing and deprescribing should also include antipsychotics. Especially, instead of using antipsychotics for their sedative-hypnotic properties in anxiety and adjustment disorders and insomnia, non-pharmacological interventions or use of antihistamines could serve as better alternatives and should be tried first. Furthermore, deprescribing efforts seem especially relevant, given the high proportion of long-term users with diagnoses of dementia, adjustment disorders, and no relevant diagnoses for antipsychotic use. Finally, continuous side effect monitoring during antipsychotic treatment is standard of care in psychiatry and should apply to all antipsychotic users. Therefore, monitoring should be directed to the group of users in other medical specialties and those treated in primary care, consisting of 41% of antipsychotic-treated individuals in 2018, and 49% of new antipsychotic users. Given the substantial number of off-label users, the potential side-effects of antipsychotics become even more relevant. Consequences of off-label and/or low-dose use of antipsychotics should be investigated, especially of quetiapine, which was by far the most used antipsychotic regardless of the diagnostic group.

In conclusion, antipsychotic use has increased in both severe and non-severe mental disorders in Denmark over the past two decades. More than one-third of all antipsychotic users had no psychiatric, neurological or cancer diagnoses as possible indications for antipsychotic therapy. Health insurance data indicate that a considerable proportion of antipsychotics is prescribed in general practice and that long-term prescribing for adjustment disorders and patients without relevant indications for antipsychotic use occurs in a concerningly large subgroup. Reasons for the considerable off-label use and its risk–benefit ratio warrant further investigation.

## Data Availability

The data used for this study are not publicly available, but can be obtained by application to The Danish Health Data Authority (www.sundhedsdatastyrelsen.dk).

## Supplementary material

For supplementary material accompanying this paper visit http://dx.doi.org/10.1017/S2045796021000159. click here to view supplementary material
