# Supplementary material for: Use of antipsychotics in Denmark 1997–2018: a nation-wide drug utilisation study with focus on off-label use and associated diagnoses
Source: Epidemiol Psychiatr Sci. 2021 Apr 6;30:e28. doi: 10.1017/S2045796021000159 (PMC8170176; doi:10.1017/S2045796021000159)

**SUPPLEMENTARY MATERIAL**

**Use of Antipsychotics in Denmark 1997-2018: A Nation-wide Drug Utilization Study with Focus on Off-label Use and Associated Diagnoses**

Højlund M, Andersen JH, Andersen K, Correll CU, Hallas J. 2021

**Contents**

[Appendix 1: Overview of data sources 2](#_Toc64614188)

[Appendix 2: ICD-codes used for diagnostic classification 3](#_Toc64614189)

[Appendix 3: ICD-10 and ATC codes used for covariate assessment in group 6 4](#_Toc64614190)

[Supplementary Table 1: Sensitivity analysis extending the assessment period for incident users 5](#_Toc64614191)

[Supplementary Table 2: Users by commonly used antipsychotics and diagnostic groups, Denmark, 2018 6](#_Toc64614192)

[Supplementary Table 3: Characteristics of antipsychotic users without psychiatric, neurological or cancer diagnoses in Denmark 2018 (n=46,923) 7](#_Toc64614193)

[Supplementary Table 4: Characteristics of antipsychotic users without psychiatric, neurological or cancer diagnoses in Denmark 2018 for the 10 most commonly used antipsychotics 8](#_Toc64614194)

[Supplementary Figure 1: Development in all users overall and by diagnostic subgroups, Denmark 1997-2018 10](#_Toc64614195)

[Supplementary Figure 2: Development in prevalent users overall and by subgroups, Denmark 1997-2018 11](#_Toc64614196)

[Supplementary Figure 3: Development in incident users overall and by subgroup, Denmark 1997-2018 12](#_Toc64614197)

# Appendix 1: Overview of data sources

| **Data source** | **Coverage** | **Content** | **Obtained information** | **Notes** |
| --- | --- | --- | --- | --- |
| Danish Register of Medical Product Statistics (DRMPS) | From 1995 | Information on all prescriptions dispensed at Danish community pharmacies, including preparation, amount, and strength. | Prescriptions for antipsychotics.  Prescriptions for other psychotropic medications.  Prescriptions for other drugs for assessment of non-psychiatric comorbidities. | In-hospital use is not covered by DRMPS. Indications not recorded in DRMPS. |
| Danish National Patient Register (DNPR) | Admissions: From 1977  Outpatient contacts: From 1995 | Information on admissions and outpatient contacts to all public Danish hospitals. | Diagnoses of psychiatric or neurological disorders.  Diagnoses of malignant neoplasms.  Diagnoses of other medical conditions for assessment of co-morbidities. | Private hospitals only accounts for a negligible proportion of health care in the included disease categories. |
| National Health Insurance Service Register (NHISR) | From 1990 | Information on all contacts to general practitioners and practicing specialists (e.g. practicing psychiatrists).  Based on invoices to regional health administrations (date and type of service). | Health care contacts to:   - General practitioners - Office-based psychiatrists - Office-based neurologists | Private practitioners only accounts for a negligible proportion of health care in the included disease categories. |
| Danish Civil Registration Register (DCRS) | Established in 1968  (covers the entire population) | Information on vital status and civil registration numbers for residents in Denmark. | Civil registration number for register-linkage | Unique identifier (civil registration number) assigned to all Danish residents upon birth or immigration allowing linkage of registers. |

# Appendix 2: ICD-codes used for diagnostic classification

| **Group** | **Subgroups** | **Codes** |
| --- | --- | --- |
| Severe or chronic mental disorders  (group 1) | Schizophrenia and schizoaffective disorder | ICD-10: F20, 25  ICD-8: 295.xx |
|  | Mania and bipolar affective disorder | ICD-10: F30-31  ICD-8: 296.xx |
|  | Other psychotic disorders | ICD-10: F22-24, 26-29  ICD-8: 297.xx-299.xx |
| Chronic mental disorders  (group 2) | Dementias | ICD-10: F00-03, G30-31  ICD-8: 290.xx |
|  | Mental retardation and autism | ICD-10: F70-79, 84  ICD-8: 311.xx-315.xx |
| Other mental disorders  (group 3) | Organic mental disorders (excl. dementia) | F04-09 |
|  | Psychoactive substance use | F10-19 |
|  | Schizotypal disorder | F21 |
|  | Affective disorders (excl. bipolar affective disorder) | F32-33, 34, 39 |
|  | Neurotic or stress-related disorders | F40-49 |
|  | Other behavioral disorders | F50-59 |
|  | Disorder of adult personality and behavior | F60-69 |
|  | Developmental disorders (excl. autism) | F80-83, 85-89 |
|  | Behavioral and emotional disorders  (e.g. hyperkinetic disorder) | F90-98 |
|  | Unspecified mental disorder | F99 |
| Selected diseases of the nervous system  (group 4) | Encephalitis, myelitis, and encephalomyelitis | G04-05 |
|  | Huntington disease | G10 |
|  | Parkinson disease | G20 |
|  | Multiple sclerosis | G35 |
|  | Episodic and paroxysmal disorders  (e.g. epilepsy, migraine, sleep disorders) | G40-47 |
|  | Other disorders of the nervous system  (e.g. hydrocephalus) | G90-99 |
| Diagnosis of cancer  (group 5) | Malignant neoplasms (excl. non-melanoma skin cancer) | C00-43, 45-97 |
| ICD: World Health Organization International Statistical Classification of Diseases and Related Health Problems 8^th^/10^th^ revision (10^th^ revision: icd.who.int/browse10/2019/en) | | |

# Appendix 3: ICD-10 and ATC codes used for covariate assessment in group 6

| **Co-medications** |  |
| --- | --- |
| Anti-dementia drugs | ATC: N06D |
| Antidepressants | ATC: N06A |
| Anxiolytics | ATC: N05B |
| Drugs used in alcohol dependence | ATC: N07BB |
| Drugs used in opioid dependence | ATC: N07BC |
| Hypnotics | ATC: N05C |
| Mood stabilizers | ATC: N03AX, N05AN01 |
| Psychostimulants | ATC: N06B |
| **Co-morbidities** |  |
| Chronic obstructive pulmonary disease | ICD-10: J40-44  ATC: R03 |
| Hypertension | ICD-10: I10-15, H350H, I674, O10  ATC: C03A, C08C, C09A |
| Alcoholism-related disease | ICD-10: E244, E529A, F10, G312, G405, G621, G721, I426, K292, K70, K860, O354, P043, T519, Z502, Z714, Z721  ATC: N07BB |
| Ischemic heart disease | ICD-10: I200-201, I208-214, I219, I22-23, I241, I252  ATC: N02BA, C01DA |
| Diabetes | ICD-10: E10-14, E891, G590, G632, G730, G990C, H280, H360, I729A, M142, N083, O240-243  ATC: A10 |
| ATC: Anatomic Therapeutic Chemical Classification System (who.int/classifications/atcddd/en), ICD-10: World Health Organization International Statistical Classification of Diseases and Related Health Problems 10^th^ revision (icd.who.int/browse10/2019/en) | |

# Supplementary Table 1: Sensitivity analysis extending the assessment period for incident users

|  | **Assessment window** | | | | | |
| --- | --- | --- | --- | --- | --- | --- |
|  | **1 year** | | **2 years** | | **5 years** | |
| **ICD-10 diagnosis** | **N** | **%^a^** | **N** | **% ^a^** | **N** | **% ^a^** |
| All incident users | 37,478 | 100 | 33,868 | 100 | 29,634 | 100 |
| Severe mental disorders | 5,469 | 15 | 4,246 | 13 | 2,917 | 9.8 |
| Chronic mental disorders | 4,082 | 11 | 3,778 | 11 | 3,452 | 12 |
| Other mental disorders | 9,850 | 26 | 9,268 | 27 | 8,353 | 28 |
| Selected diseases of the nervous system | 1,386 | 3.7 | 1,335 | 3.9 | 1,278 | 4.3 |
| Advanced cancers | 2,217 | 5.9 | 2,190 | 6.5 | 2,152 | 7.3 |
| No relevant diagnoses | 14,474 | 39 | 13,051 | 39 | 11,482 | 39 |

Notes: ^a^Percentage of all incident users.

Abbreviations: ICD-10: World Health Organization International Statistical Classification of Diseases and Related Health Problems 10^th^ revision (icd.who.int/browse10/2019/en).

# Supplementary Table 2: Users by commonly used antipsychotics and diagnostic groups, Denmark, 2018

ARI: Aripiprazole, CHL: Chlorprothixene, CLO: Clozapine, FLU: Flupentixol, HAL: Haloperidol, LEV: Levomepromazine, OLA: Olanzapine, QUE: Quetiapine, RIS: Risperidone, ZUC: Zuclopenthixol.

|  | **CLO** | **ZUC** | **ARI** | **OLA** | **RIS** | **CHL** | **QUE** | **LEV** | **FLU** | **HAL** |
| --- | --- | --- | --- | --- | --- | --- | --- | --- | --- | --- |
| **All users** |  |  |  |  |  |  |  |  |  |  |
| All groups | 3,403 (100) | 4,224 (100) | 12,357 (100) | 17,554 (100) | 16,056 (100) | 14,028 (100) | 64,946 (100) | 4,017 (100) | 3,809 (100) | 7,963 (100) |
| Severe mental disorders | 3,113 (91) | 3,061 (72) | 8,033 (65) | 10,671 (61) | 6,707 (42) | 3,895 (28) | 17,578 (27) | 811 (20) | 497 (13) | 848 (11) |
| Chronic mental disorders | 130 (3.8) | 364 (8.6) | 1,159 (9.4) | 1,624 (9.3) | 4,175 (26) | 994 (7.1) | 5,720 (8.8) | 337 (8.4) | 140 (3.7) | 1,482 (19) |
| Other mental disorders | 21 (.62) | 87 (2.1) | 1,236 (10) | 1,937 (11) | 1,714 (11) | 2,276 (16) | 12,786 (20) | 251 (6.2) | 281 (7.4) | 1,219 (15) |
| Neurological diagnosis only | 52 (1.5) | 14 (.33) | 77 (.62) | 318 (1.8) | 138 (.86) | 169 (1.2) | 962 (1.5) | 234 (5.8) | 69 (1.8) | 714 (9) |
| Cancer diagnosis only | - | 16 (.38) | 5 (.04) | 370 (2.1) | 103 (.64) | 144 (1) | 453 (.7) | 82 (2) | 61 (1.6) | 1,816 (23) |
| No relevant diagnosis | 81 (2.4) | 679 (16) | 1,843 (15) | 2,634 (15) | 3,219 (20) | 6,550 (47) | 27,447 (42) | 2,302 (57) | 2,761 (72) | 1,884 (24) |
| **Prevalent users** |  |  |  |  |  |  |  |  |  |  |
| All groups | 3,253 (100) | 3,987 (100) | 10,167 (100) | 13,983 (100) | 11,976 (100) | 10,519 (100) | 45,037 (100) | 3,119 (100) | 3,076 (100) | 1,683 (100) |
| Severe mental disorders | 3,029 (93) | 2,929 (73) | 6,959 (68) | 9,474 (68) | 5,832 (49) | 3,477 (33) | 15,069 (33) | 761 (24) | 460 (15) | 703 (42) |
| Chronic mental disorders | 108 (3.3) | 341 (8.6) | 947 (9.3) | 1,312 (9.4) | 2,929 (24) | 815 (7.7) | 4,091 (9.1) | 307 (9.8) | 119 (3.9) | 442 (26) |
| Other mental disorders | 15 (.46) | 63 (1.6) | 743 (7.3) | 968 (6.9) | 827 (6.9) | 1,230 (12) | 6,240 (14) | 138 (4.4) | 175 (5.7) | 143 (8.5) |
| Neurological diagnosis only | 39 (1.2) | 14 (.35) | 59 (.58) | 96 (.69) | 68 (.57) | 122 (1.2) | 605 (1.3) | 78 (2.5) | 57 (1.9) | 54 (3.2) |
| Cancer diagnosis only | - | 16 (.4) | 5 (.049) | 73 (.52) | 40 (.33) | 110 (1) | 252 (.56) | 55 (1.8) | 50 (1.6) | 86 (5.1) |
| No relevant diagnosis | 58 (1.8) | 624 (16) | 1,454 (14) | 2,060 (15) | 2,280 (19) | 4,765 (45) | 18,780 (42) | 1,780 (57) | 2,215 (72) | 255 (15) |
| **Incident users** |  |  |  |  |  |  |  |  |  |  |
| All groups | 150 (100) | 237 (100) | 2,190 (100) | 3,571 (100) | 4,080 (100) | 3,509 (100) | 19,909 (100) | 898 (100) | 733 (100) | 6,280 (100) |
| Severe mental disorders | 84 (56) | 132 (56) | 1,074 (49) | 1,197 (34) | 875 (21) | 418 (12) | 2,509 (13) | 50 (5.6) | 37 (5) | 145 (2.3) |
| Chronic mental disorders | 22 (15) | 23 (9.7) | 212 (9.7) | 312 (8.7) | 1,246 (31) | 179 (5.1) | 1,629 (8.2) | 30 (3.3) | 21 (2.9) | 1,040 (17) |
| Other mental disorders | 6 (4) | 24 (10) | 493 (23) | 969 (27) | 887 (22) | 1,046 (30) | 6,546 (33) | 113 (13) | 106 (14) | 1,076 (17) |
| Neurological diagnosis only | 13 (8.7) | - | 18 (.82) | 222 (6.2) | 70 (1.7) | 47 (1.3) | 357 (1.8) | 156 (17) | 12 (1.6) | 660 (11) |
| Cancer diagnosis only | - | - | - | 297 (8.3) | 63 (1.5) | 34 (.97) | 201 (1) | 27 (3) | 11 (1.5) | 1,730 (28) |
| No relevant diagnosis | 23 (15) | 55 (23) | 389 (18) | 574 (16) | 939 (23) | 1,785 (51) | 8,667 (44) | 522 (58) | 546 (74) | 1,629 (26) |

# Supplementary Table 3: Characteristics of antipsychotic users without psychiatric, neurological or cancer diagnoses in Denmark 2018 (n=46,923)

| **Characteristic** | **N** | **%** |
| --- | --- | --- |
| **Age** |  |  |
| 0-12 years | 65 | 0.1 |
| 13-17 years | 236 | 0.5 |
| 18-24 years | 2,286 | 4.9 |
| 25-44 years | 11,759 | 25.1 |
| 45-64 years | 17,669 | 37.7 |
| 65-79 years | 9,445 | 20.1 |
| 80+ years | 5,463 | 11.6 |
| **Sex** |  |  |
| Female | 26,917 | 57.4 |
| **Antipsychotic drugs** |  |  |
| Quetiapine | 27,447 | 58.5 |
| Chlorprothixene | 6,550 | 14.0 |
| Risperidone | 3,219 | 6.9 |
| Flupentixol | 2,761 | 5.9 |
| Olanzapine | 2,634 | 5.6 |
| Levomepromazine | 2,302 | 4.9 |
| Haloperidol | 1,884 | 4.0 |
| Aripiprazole | 1,843 | 3.9 |
| Zuclopenthixol | 679 | 1.4 |
| Clozapine | 81 | 0.2 |
| **Number of antipsychotic prescriptions** |  |  |
| 1 | 13,368 | 28.5 |
| 2 | 5,433 | 11.6 |
| 3-5 | 14,415 | 30.7 |
| >5 | 13,707 | 29.2 |
| **Number of antipsychotic drugs** |  |  |
| 1 | 43,620 | 93.0 |
| 2 | 3,009 | 6.4 |
| >2 | 294 | 0.6 |
| **Total amount redeemed** |  |  |
| ≤90 DDD | 37,640 | 80.2 |
| 91-180 DDD | 4,635 | 9.9 |
| 181-365 DDD | 2,984 | 6.4 |
| >365 DDD | 1,633 | 3.5 |
| **Concurrent use of psychotropic co-medication** |  |  |
| Antidepressants (N06A) | 24,064 | 51.3 |
| Hypnotics (N05C) | 8,793 | 18.7 |
| Mood stabilizers (N03AX+N05AN01) | 7,499 | 16.0 |
| Anxiolytics (N05B) | 5,804 | 12.4 |
| Psychostimulants (N06B) | 2,535 | 5.4 |
| Drugs for alcohol dependence (N07BB) | 829 | 1.8 |
| Drugs for opioid dependence (N07BC) | 591 | 1.3 |
| Anti-dementia drugs (N06D) | 270 | 0.6 |
| **Somatic comorbidity** |  |  |
| Chronic obstructive pulmonary disease | 20,999 | 44.8 |
| Hypertension | 19,853 | 42.3 |
| Alcoholism-related disease | 9,199 | 19.6 |
| Ischemic heart disease | 8,142 | 17.4 |
| Diabetes | 5,793 | 12.3 |
| **First prescriber*** |  |  |
| Psychiatrist | 1,721 | 11.9 |
| General practitioner | 9,434 | 65.2 |
| Other practicing specialist | 134 | 0.9 |
| Hospital | 467 | 3.2 |
| Psychiatric hospital | 247 | 1.7 |
| No information | 2,718 | 18.8 |
| **Healthcare utilization*** |  |  |
| Contact with GP only | 11,527 | 79.6 |
| Contact with practicing psychiatrist | 2,383 | 16.5 |
| Contact with other practicing specialist | 309 | 2.1 |
| No information | 255 | 1.8 |
| *Among incident users in 2018 (n = 14,474) |  |  |

# Supplementary Table 4: Characteristics of antipsychotic users without psychiatric, neurological or cancer diagnoses in Denmark 2018 for the 10 most commonly used antipsychotics

ARI: Aripiprazole, CHL: Chlorprothixene, CLO: Clozapine, FLU: Flupentixol, HAL: Haloperidol, LEV: Levomepromazine, OLA: Olanzapine, QUE: Quetiapine, RIS: Risperidone, ZUC: Zuclopenthixol. (Cells filled with – means few or zero observations and have been blinded due to confidentiality issues).

|  | **QUE** | **OLA** | **RIS** | **CHL** | **ARI** |
| --- | --- | --- | --- | --- | --- |
|  | n = 27,447 | n = 2,634 | n = 3,219 | n = 6,550 | n = 1,843 |
| **Age, median (IQR)** | 50 (38-62) | 58 (46-72) | 59 (43-79) | 54 (43-66) | 44 (30-56) |
| **Sex** |  |  |  |  |  |
| Female | 15,485 (56.4%) | 1,288 (48.9%) | 1,677 (52.1%) | 3,684 (56.2%) | 1,018 (55.2%) |
| **Starting year, median (IQR)** | 2014 (2009-2017) | 2011 (2003-2016) | 2013 (2005-2017) | 2010 (2003-2015) | 2012 (2006-2016) |
| **Number of antipsychotic prescriptions** |  |  |  |  |  |
| 1 | 7,581 (27.6%) | 372 (14.1%) | 517 (16.1%) | 1,835 (28.0%) | 200 (10.9%) |
| 2 | 3,317 (12.1%) | 263 (10.0%) | 348 (10.8%) | 706 (10.8%) | 197 (10.7%) |
| 3-5 | 8,827 (32.2%) | 729 (27.7%) | 738 (22.9%) | 1,755 (26.8%) | 552 (30.0%) |
| >5 | 7,722 (28.1%) | 1,270 (48.2%) | 1,616 (50.2%) | 2,254 (34.4%) | 894 (48.5%) |
| **Total amount redeemed** |  |  |  |  |  |
| ≤90 DDD | 22,575 (82.2%) | 982 (37.3%) | 2,203 (68.4%) | 5,475 (83.6%) | 648 (35.2%) |
| 91-180 DDD | 2,365 (8.6%) | 628 (23.8%) | 539 (16.7%) | 552 (8.4%) | 538 (29.2%) |
| 181-365 DDD | 1,593 (5.8%) | 553 (21.0%) | 350 (10.9%) | 316 (4.8%) | 388 (21.1%) |
| >365 DDD | 900 (3.3%) | 466 (17.7%) | 122 (3.8%) | 201 (3.1%) | 264 (14.3%) |
| **First prescriber** |  |  |  |  |  |
| Psychiatrist | 1,161 (13.4%) | 119 (20.7%) | 143 (15.2%) | 174 (9.7%) | 182 (46.8%) |
| General practitioner | 5,848 (67.5%) | 287 (50.0%) | 512 (54.5%) | 1,284 (71.9%) | 81 (20.8%) |
| Hospital | 100 (1.2%) | 53 (9.2%) | 45 (4.8%) | 8 (0.4%) | - |
| **Healthcare utilization** |  |  |  |  |  |
| Contact with GP only | 6,775 (78.2%) | 412 (71.8%) | 734 (78.2%) | 1,429 (80.1%) | 167 (42.9%) |
| Contact with practicing psychiatrist | 1,624 (18.7%) | 145 (25.3%) | 181 (19.3%) | 288 (16.1%) | 203 (52.2%) |

**Supplementary Table 4 (cont.)**

|  | **HAL** | **ZUC** | **LEV** | **FLU** | **CLO** |
| --- | --- | --- | --- | --- | --- |
|  | n = 1,884 | n = 679 | n = 2,302 | n = 2,761 | n = 81 |
| **Age, median (IQR)** | 87 (79-93) | 67 (56-76) | 67 (58-76) | 66 (53-76) | 71 (56-77) |
| **Sex** |  |  |  |  |  |
| Female | 1,128 (59.9%) | 1,547 (67.2%) | 425 (62.6%) | 1,929 (69.9%) | 36 (44.4%) |
| **Starting year, median (IQR)** | 2018 (2018-2018) | 2003 (1995-2014) | 1995 (1995-2002) | 2003 (1995-2013) | 2013 (2002-2017) |
| **Number of antipsychotic prescriptions** |  |  |  |  |  |
| 1 | 1,316 (69.9%) | 656 (28.5%) | 64 (9.4%) | 608 (22.0%) | 17 (21.0%) |
| 2 | 263 (14.0%) | 274 (11.9%) | 48 (7.1%) | 296 (10.7%) | 15 (18.5%) |
| 3-5 | 174 (9.2%) | 687 (29.8%) | 255 (37.6%) | 1,181 (42.8%) | 23 (28.4%) |
| >5 | 131 (7.0%) | 685 (29.8%) | 312 (45.9%) | 676 (24.5%) | 26 (32.1%) |
| **Total amount redeemed** |  |  |  |  |  |
| ≤90 DDD | 1,707 (90.6%) | 2,131 (92.6%) | 464 (68.3%) | 2,410 (87.3%) | 59 (72.8%) |
| 91-180 DDD | 91 (4.8%) | 81 (3.5%) | 103 (15.2%) | 247 (8.9%) | 7 (8.6%) |
| 181-365 DDD | 52 (2.8%) | 46 (2.0%) | 69 (10.2%) | 78 (2.8%) | 9 (11.1%) |
| >365 DDD | 30 (1.6%) | 41 (1.8%) | 40 (5.9%) | 24 (0.9%) | 6 (7.4%) |
| **First prescriber** |  |  |  |  |  |
| Psychiatrist | - | 18 (3.4%) | 7 (12.7%) | 109 (20.0%) | - |
| General practitioner | 1,043 (64.0%) | 305 (58.4%) | 26 (47.3%) | 368 (67.4%) | 10 (43.5%) |
| Hospital | 261 (16.0%) | 21 (4.0%) | - | - | - |
| **Healthcare utilization** |  |  |  |  |  |
| Contact with GP only | 1,562 (95.9%) | 397 (76.1%) | 43 (78.2%) | 393 (72.0%) | 18 (78.3%) |
| Contact with practicing psychiatrist | 5 (0.3%) | 28 (5.4%) | 10 (18.2%) | 140 (25.6%) | - |

# Supplementary Figure 1: Development in all users overall and by diagnostic subgroups, Denmark 1997-2018

A) Groups 1 to 6, B) Severe and chronic mental disorders, C) Other mental disorders, D) Neurological disorders

**
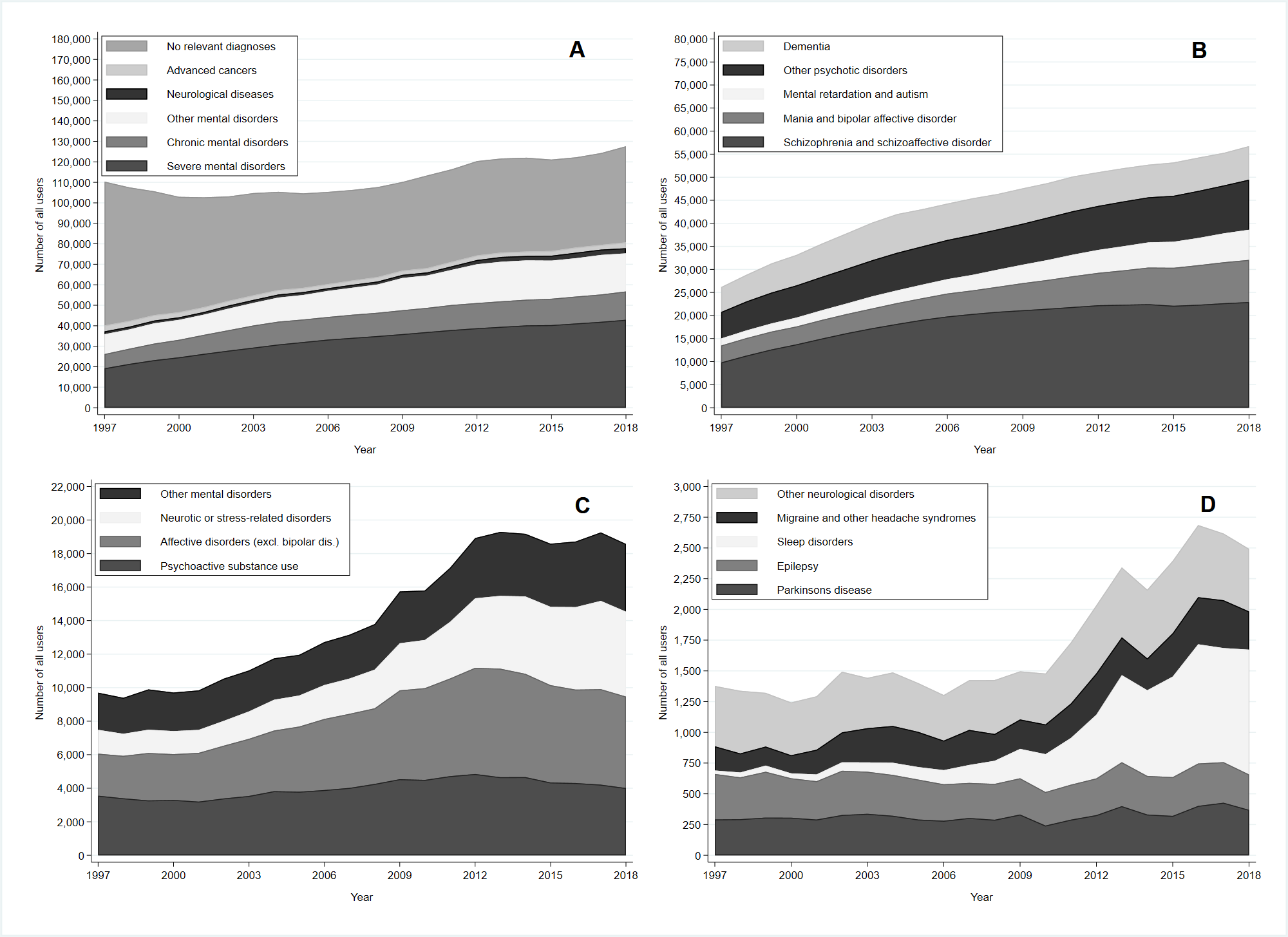
**

# Supplementary Figure 2: Development in prevalent users overall and by subgroups, Denmark 1997-2018

A) Groups 1 to 6, B) Severe and chronic mental disorders, C) Other mental disorders, D) Neurological disorders


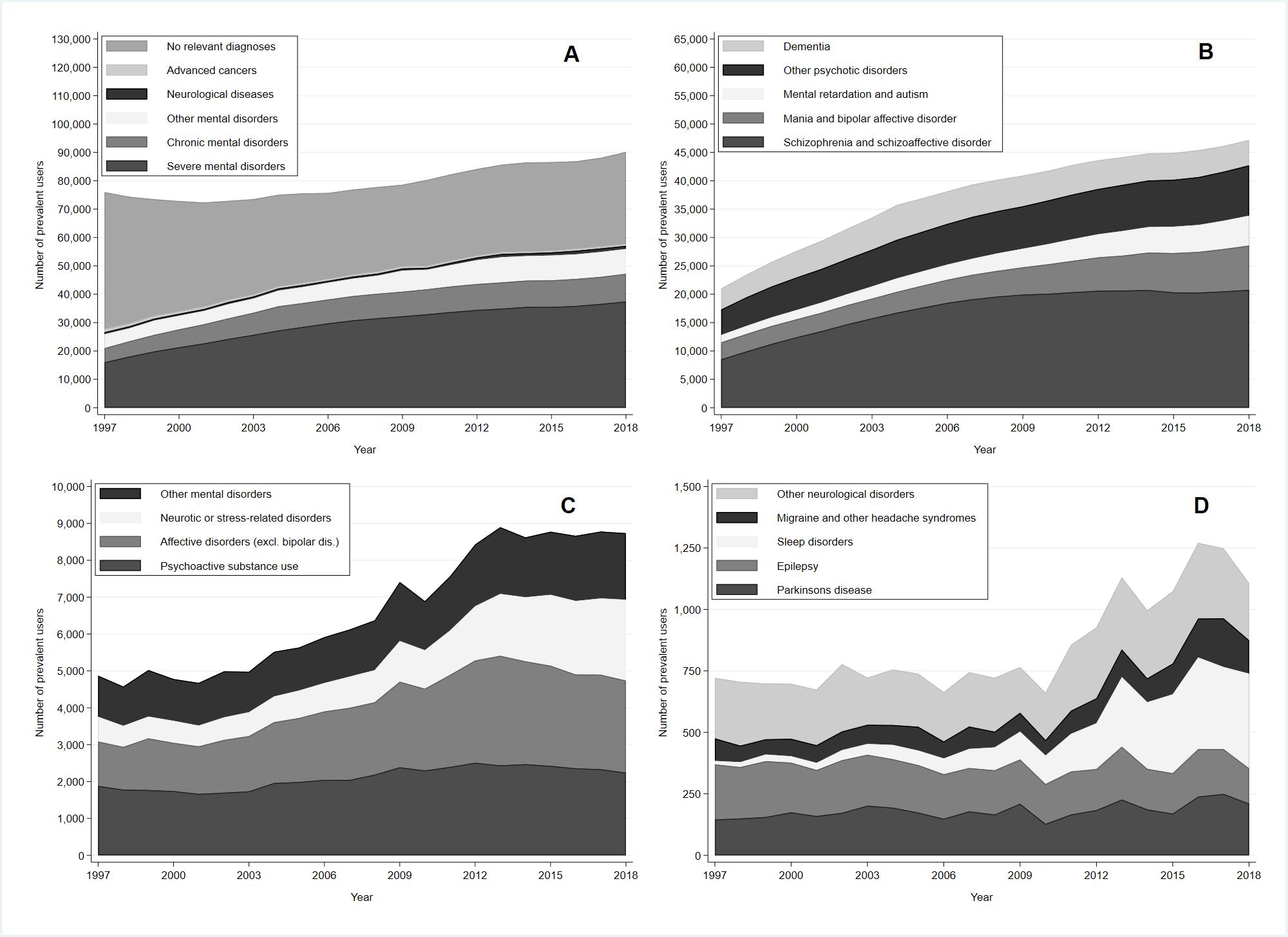


# Supplementary Figure 3: Development in incident users overall and by subgroup, Denmark 1997-2018

A) Groups 1 to 6, B) Severe and chronic mental disorders, C) Other mental disorders, D) Neurological disorders


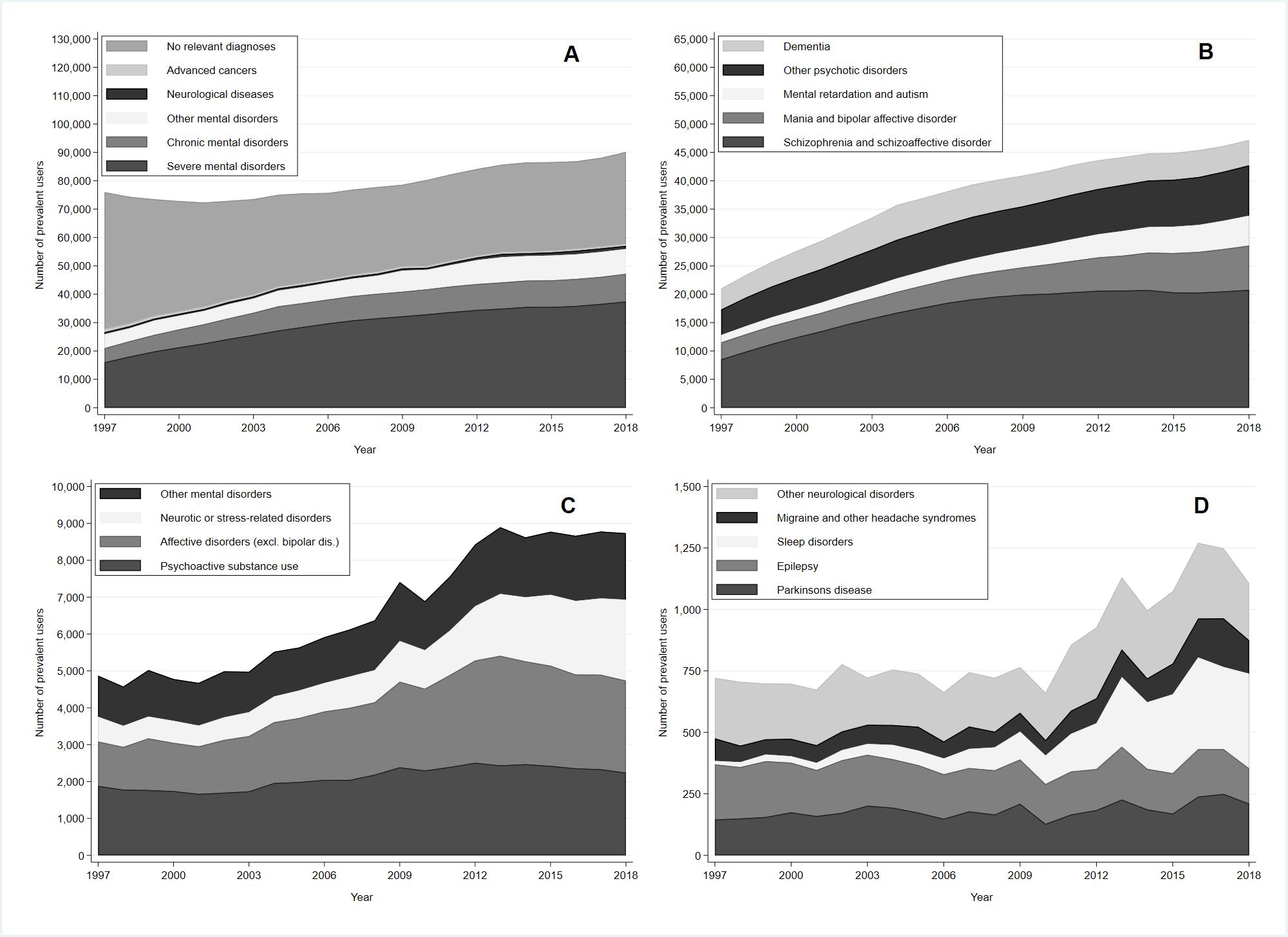

Supplement: Supplementary file 1 [file S2045796021000159sup001.docx]
